# Gender differences in all-cause and cardiovascular mortality among US adults: from NHANES 2005–2018

**DOI:** 10.3389/fcvm.2024.1283132

**Published:** 2024-02-14

**Authors:** Ying Lv, Xiaodi Cao, Kai Yu, Jie Pu, Zhiguo Tang, Na Wei, Junkui Wang, Fuqiang Liu, Shangjian Li

**Affiliations:** ^1^ Department of Cardiology, Shaanxi Provincial People’s Hospital, Xi’an, Shaanxi, China; ^2^ Department of Cardiology, Jiangsu Provincial People’s Hospital and The First Affiliated Hospital of Nanjing Medical University, Nanjing, Jiangsu, China; ^3^ Department of Cardiology, Pucheng County Hospital, Weinan, Shaanxi, China

**Keywords:** gender difference, all-cause mortality, cardiovascular mortality, mediation analysis, uric acid

## Abstract

**Background:**

Gender disparities in mortality have drawn great interest, with previous studies identifying various biological, social, and behavioral factors contributing to the observed gender differences. This study aims to identify the sources of gender disparities in mortality rates and quantify the extent to which these factors mediate the gender differences in all-cause mortality.

**Methods:**

Data from the National Health and Nutrition Examination Survey (NHANES) conducted between 2005 and 2018 were analyzed. A total of 38,924 participants were included in the study. Gender information, socioeconomic status, lifestyle factors, and baseline disease status were obtained through questionnaires. Blood samples were collected to assess serological indicators. All-cause and cardiovascular mortality were considered as primary and secondary outcomes, respectively.

**Results:**

The study with an average age of 50.1 ± 17.9 years. Among the participants, 50.7% were women, and 41.8% were non-Hispanic White. The median follow-up length was 87 months [Inter-Quartile Range (IQR): 47–128]. Men showed higher rates of all-cause and cardiovascular mortality compared to women in both the general population and the population with cardiovascular disease. After adjustment for potential confounders (age, race, marital status, socioeconomic status, lifestyle level, smoking status, cardiovascular disease, hypertension, diabetes and cancer), the men: women hazard ratios (HRs) for all-cause and cardiovascular mortality were 1.58 [95% Confidence Interval (CI): 1.48–1.68] and 1.60 (95%CI:1.43–1.80) in the general population. Among individuals with cardiovascular disease, the fully adjusted HR for all-cause mortality was 1.34 (95% CI: 1.20 to 1.51), and for cardiovascular mortality, the fully adjusted HRs was 1.52 (95% CI: 1.26 to 1.83). Mediation analysis revealed that uric acid levels significantly mediated the association between gender and all-cause mortality, accounting for 17.53% (95% CI: 11.0% to 23.7%) in the general population and 27.47% (95% CI: 9.0% to 13.6%) in the population with cardiovascular disease.

**Conclusions:**

The study highlights the complex interplay of biological and social factors contributing to gender disparities in mortality. Uric acid was identified as key mediators of the gender-mortality association. These findings can inform targeted interventions aimed at reducing gender disparities in mortality and promoting better public health outcomes.

## Introduction

According to Global Health 50/50, gender is defined as the socially constructed norms that impose and determine roles, relationships and positional power for individuals throughout their lifespan (1). Gender is influenced by sex, biological and physiological characteristics, in shaping the identities of both men and women. This finding suggests that women exhibit distinct distributions of certain risk factors and vulnerabilities to certain diseases compared to men. Notably, a higher mortality rate has been consistently observed among men across various age groups (2). Consequently, the disparity in health outcomes based on gender has garnered significant scholarly interest in recent decades (3).

Previous studies have identified various crucial factors that contribute to the existing gender disparities in mortality. Primarily, biological factors exert a substantial influence, as women typically possess inherent advantages that contribute to lower mortality rates. For instance, research has demonstrated that estrogen exhibits a protective effect against cardiovascular disease (CVD) (4, 5), a prominent cause of mortality. Furthermore, social and behavioral factors exert a significant influence on gender disparities in mortality. Men tend to engage in high-risk behaviors, such as smoking and excessive alcohol consumption, both of which are associated with increased mortality risks (6, 7). Additionally, socioeconomic factors play a crucial role in contributing to gender disparities in mortality. Disparities in healthcare access, education, and socioeconomic resources between men and women can substantially impact health outcomes and ultimately influence mortality rates (6). The extent to which these risk factors differences can explain the disparities in mortality risk between genders remains unclear. It is also uncertain whether interventions targeting specific modifiable risk factors can effectively address the health inequalities between men and women.

Notably, the underlying causes of gender disparities in mortality are intricate and multifaceted. The purpose of this study is to ascertain the origins of gender differences in mortality rates and investigate potential factors that can be modified, thereby providing valuable insights for public health interventions and policies aimed at reducing gender disparities in mortality and enhancing the overall health of the population.

## Materials and methods

### Study design and population

**Figure 1:**
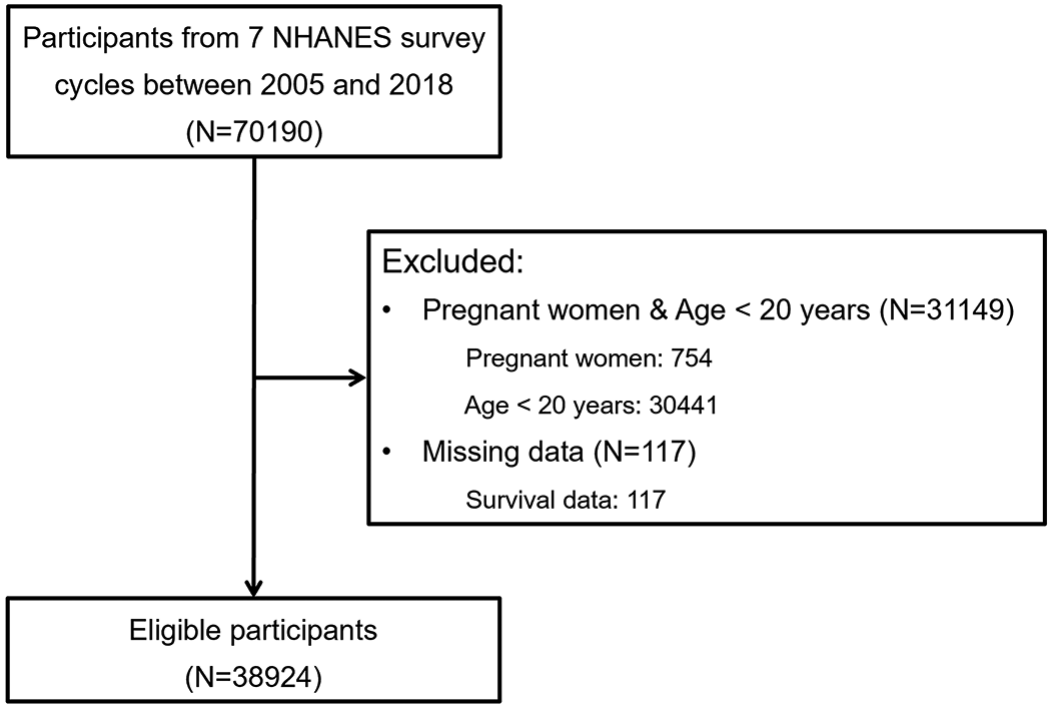
The flow chart of study participants selection.

This study utilized data from the National Health and Nutrition Examination Survey (NHANES), a population-based survey in the United States sponsored by the Centers for Disease Control and Prevention (CDC) and the National Center for Health Statistics (NCHS). NHANES is specifically designed to evaluate health and nutritional status. The study design and data collection methods have been previously documented (8). Pregnant women and individuals under the age of 20 years were excluded, since social status and biochemical indicators are not suitable for assessment in these special groups. Similar exclusion criteria have been applied in previous studies (9, 10). Finally, 38,924 participants from seven NHANES survey cycles conducted between 2005 and 2018 were included in the analysis (Figure 1). Written informed consent was obtained from all participants, and the research ethics boards of the NCHS approved all protocols. All data used in the study are publicly available.

### Baseline data collection

In this study, gender information was obtained through demographic questionnaires. Socioeconomic status (SES) and lifestyle level were categorized separately using latent class analysis (LCA) (11) to streamline the variables. Following the approach from previously published study, the variables were grouped as follows. Educational level was classified as below high school, high school, college and above (12). Household income levels were assessed using the self-reported poverty-to-income ratio (PIR) and were divided into low (≤1), medium (1–4), and high (≥4) groups (13). Health insurance was categorized into three groups: private health insurance, public health insurance only, and no health insurance (14). Occupation status was classified as high (socioeconomic index ≥ 50), low (socioeconomic index < 50, including retirees and students), and unemployed (15). SES was determined by considering education level, self-reported household income level, type of health insurance, and occupation status. SES was then categorized into three levels: high, medium, and low (Supplementary Figure S1). This approach to defining SES has been previously shown in research to be associated with mortality risks and the incidence of CVD (16).

Lifestyle level was evaluated using measures of sleep, physical activity and diet. Based on LCA, three distinct categories of lifestyle were identified: healthy, less healthy and unhealthy lifestyles (Supplementary Figure S2). A healthy sleep duration was defined as 7–9 h of sleep per night (17). The assessment of physical activity varied across different NHANES survey years in the questionnaires. From 2007 to 2018, the weekly metabolic rate (MET) (18) minutes of leisure time physical activity were calculated, while from 2005 to 2006, monthly MET minutes were calculated. In order to standardize the data, participants were divided into three categories based on their MET minutes, and the top third was considered as having a healthy level of physical activity (16). Dietary quality was assessed using the Dietary Inflammation Index (DII) (19), which was calculated based on 24-hour dietary recall data. A healthy diet was defined as having a DII score in the top two quintiles (20). Moreover, never smoking was considered a healthy behavior, and individuals were categorized as such if they reported smoking fewer than 100 cigarettes in their lifetime based on questionnaire responses (21).

Baseline disease status was determined using self-reported form, where participants were asked about their medical history. For instance, the presence of high blood pressure was determined based on participants' responses to the question: “Have you ever been told by a doctor or other health professional that you have hypertension, also known as high blood pressure?”. CVD was defined based on the definition used in the previous study (22). It encompassed the presence of coronary heart disease (self-report of myocardial infarction or heart attack), stroke (self-report of stroke), or heart failure (self-report of congestive heart failure). Additional analysis was conducted on a population with CVD due to the identification of an interaction in the risk of all-cause mortality between gender and population with history of CVD.

### Serological indicators

Blood samples were collected from participants after an overnight fast, and the serum was extracted for analysis. The samples were appropriately stored and transported to a collaborative laboratory for further testing. Detailed information regarding the data collection methods and procedures can be found on the NHANES website (http://www.cdc.gov/nchs/nhanes/). In our study, we included specific serological indicators that have been shown to exhibit differences between men and women, including high-density lipoprotein cholesterol (HDL-C), low-density lipoprotein cholesterol (LDL-C), total cholesterol, triglyceride (23), albumin (24) and uric acid (25).

### Outcome measures

The primary outcome of the study was all-cause mortality, meaning mortality from any cause. The secondary outcome was specifically cardiovascular mortality, which refers to mortality related to CVD. Mortality data, including the cause of death, were obtained from the NHANES public-related mortality files. The cause of death was recorded using the International Classification of Diseases, 10th Revision (ICD-10) coding system (26). The NCHS defined cardiovascular deaths as death caused by heart disease (ICD-10 codes I00–I09, I11, I13, and I20–I51) or cerebrovascular disease (ICD-10 codes I60–I69). This classification system has been validated by the CDC and is widely used in their reports. Participants in this study were followed from the baseline interview date until the date of death or December 31, 2019, whichever occurred first.

### Statistical analysis

Baseline characteristics of men and women were analyzed separately. The Kolmogorov-Smirnov test was employed to assess the normality of the data distribution. Two-sample Student's *t*-test was employed to compare normally distributed continuous variables between the two groups. The Wilcoxson rank-sum test, was utilized to compare non-normally distributed continuous variables. The χ^2^ test was employed to analyze categorical variables.

The Kaplan-Meier method was used to estimate the cumulative incidence and time to event of all-cause mortality in both the entire population and the population with CVD. The log-rank test was utilized to compare the survival curves between different groups. For cause-specific mortality analysis, the Fine and Gray competing risk models were used. These models allow for the estimation of the cumulative incidence probability of both cardiovascular mortality and other cause of mortality while accounting for competing events. This approach was used to assess the cumulative incidence probability of mortality in men and women (27).

**Figure 2:**
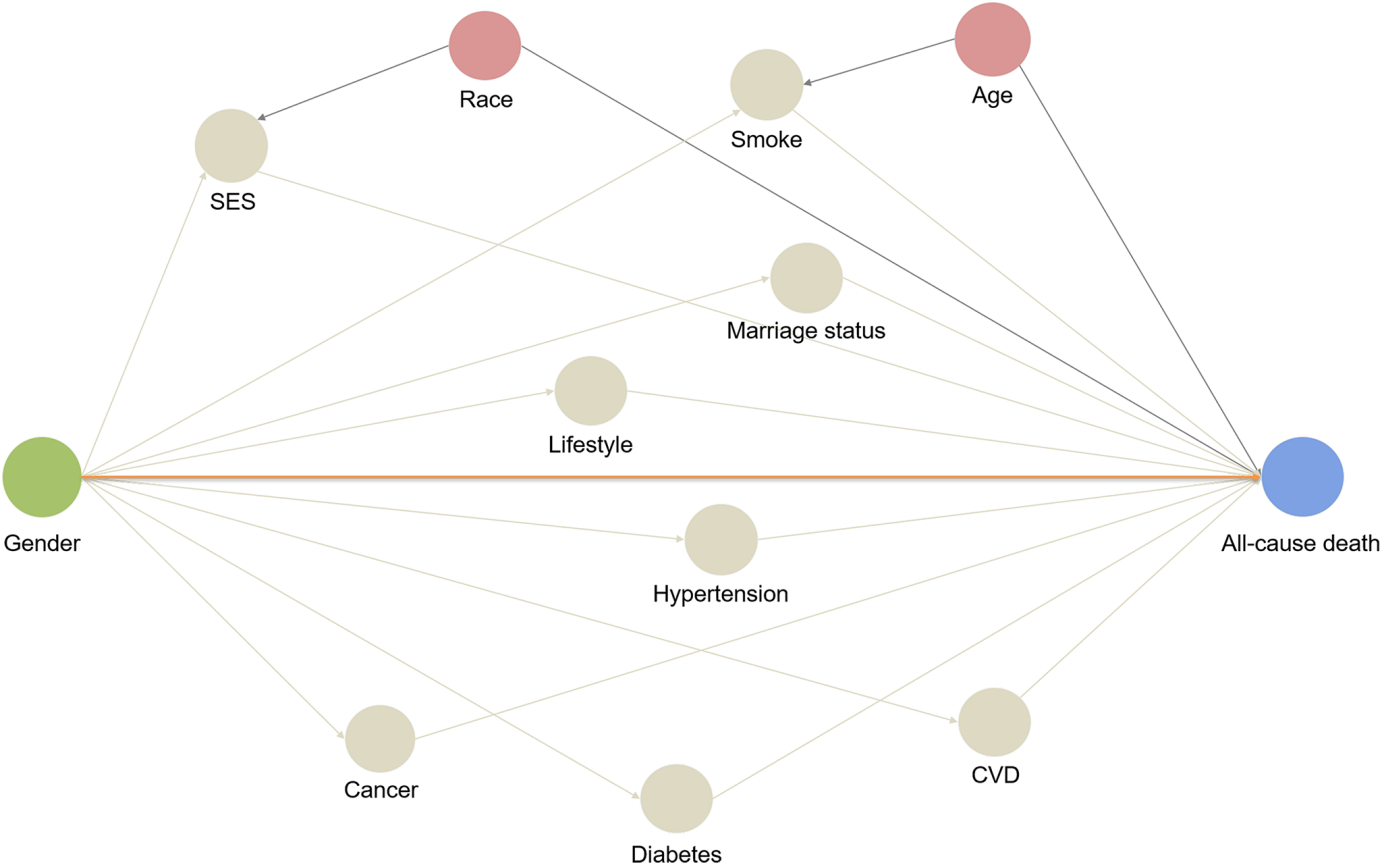
Directed acyclic graph: a hypothetical model. A directed acyclic graph represents associations between covariates and primary exposure and outcome. The orange line represents direct effect, and other lines represent biasing paths. Pink circles represent ancestors of the confounders, and the grey circles represent intermediates or mediators, which are part of a directed path.

A directed acyclic graph (DAG) (28) was constructed to identify potential confounders and mediators in the relationship between gender and mortality outcomes. The identified variables were then incorporated into the modeling strategy (Figure 2) to account for their effects. Multivariable Cox proportional hazards regression analysis was performed to estimate hazard ratios (HR) along with 95% confidence intervals (CI). These estimates were used to assess the associations between gender and the risks of all-cause mortality and cardiovascular mortality in both the entire population and individuals with CVD. The following variables were adjusted based on the DAG: confounders such as age and race, as well as mediators including marital status, SES, lifestyle level, smoking status, CVD, hypertension, diabetes and cancer. By adjusting for these variables, the aim was to investigate the actual difference in mortality risk between men and women, while considering potential confounding and mediating factors. Multicollinearity tests were conducted to assess the correlation between independent variables. Additionally, the interaction between gender and the aforementioned covariates was explored to determine which factors contributed to the gender difference in mortality. Subgroup analysis was conducted, and differences between groups were considered to be present if the interaction *P*-value was <0.05. This allowed for the identification of specific factors that interacted with gender and influenced mortality outcomes.

In addition, to explore potential mediators of gender differences in all-cause mortality, we conducted mediation analysis in both the total population and individuals with CVD. Mediators were variables that help explain the relationship between the independent variable (gender) and the outcome variable (all-cause mortality) (29, 30). We utilized the R package “Regmedint” to perform mediation analysis with survival data (31). Mediation analysis allows for the examination of the association between the exposure (gender) and all-cause mortality by decomposing it into two distinct components: the natural direct effect (NDE) and the natural indirect effect (NIE). The NDE represents the direct impact of the exposure on the outcome, independent of any potential mediators. While the NIE reflects the influence of the exposure that operates through the mediator. The proportion mediation (PM) is calculated using the formula: PM=NIE/TE (32). Based on previous definitions in the research, key mediators were defined as those that exhibited a significant mediating effect and had a PM greater than 10% (33).

All statistical analyses were conducted using R version 4.2.3. A two-sided *P*-value of less than 0.05 was considered statistically significant. The Schoenfeld test was employed to assess the assumption of proportional hazards (PH). Additionally, we assessed multicollinearity using the variance inflation factor (VIF). We observed that no VIF was greater than or equal to 5. The proportion of missing data for all covariates was less than 17%. To handle missing patient-reported data, multiple imputation was performed using the Multivariate Imputation Chain Equation (MICE) algorithm, implemented in the R package (34). This method allows for the imputation of missing values based on the relationships between variables, helping to mitigate bias and maintain statistical power.

## Results

### Population characteristics

The baseline characteristics of the 38,924 participants were summarized in Table 1. The average age of the participants was 50.1 ± 17.9 years, with 50.7% of them being women and 41.8% were non-Hispanic White. Men had lower Body Mass Index (BMI) compared to women (28.7 ± 6.1 kg/m^2^ *vs.* 29.6 ± 7.6 kg/m^2^, *P *< 0.001). Men were more likely to be married, smoke cigarettes, have higher SES, and lead healthy lifestyle compared to women. The prevalence of CVD was found to be higher in men. Furthermore, men had higher baseline levels of uric acid, albumin, total protein, and triglycerides, while women had higher levels of HDL-C, total cholesterol, and LDL-C.

**Table 1:** Baseline characteristics of men and Women: the United States, 2005–2018.

Characteristics	Men	Women	*P-*value
	*N* = 19,199	*N* = 19,725	
Age, yrs	49.9 ± 18.0	50.3 ± 17.9	0.052
SBP, mmHg	125.9 ± 17.5	123.3 ± 20.1	<0.001
DBP, mmHg	71.5 ± 13.0	68.8 ± 12.7	<0.001
Waist circumference, cm	101.3 ± 15.8	97.6 ± 16.9	<0.001
BMI, kg/m^2^	28.7 ± 6.1	29.6 ± 7.6	<0.001
DII score	−0.1 [−2.1, 0.8]	0.1 [−0.9, 1.5]	<0.001
Sleep disorder, %	21.3	29.0	<0.001
Race, %			<0.001
Mexican American	15.6	15.3	
Other Hispanic	8.8	10.4	
Non-Hispanic White	42.6	41.0	
Non-Hispanic Black	21.5	21.8	
Other Race—Including Multi Racial	11.5	11.4	
Marital Status, %			<0.001
Married	59.8	48.9	
Widowed/divorced/separated	17.9	31.2	
Unmarried	22.3	19.9	
SES, %			<0.001
High	26.0	24.5	
Median	45.3	43.6	
Low	28.7	31.9	
Lifestyle, %			<0.001
Healthy	47.1	32.1	
Less healthy	29.8	39.2	
Unhealthy	23.0	28.8	
Cigarette smoking, %			<0.001
Never	45.6	64.7	
Former	30.1	18.2	
Current	24.4	17.1	
Hypertension, %	35.5	37.2	<0.001
Diabetes, %	14.1	13.1	0.004
CVD, %	10.5	8.0	<0.001
Cancer, %	9.3	10.1	0.004
Blood test			
Uric acid, mg/dl	6.1 ± 1.3	4.9 ± 1.3	<0.001
Albumin, g/L	43.0 ± 3.7	41.5 ± 3.6	<0.001
Total protein, g/L	71.9 ± 4.7	71.3 ± 4.6	<0.001
Triglyceride, mg/dl	129.0 [86.0, 203.0]	114.0 [77.0, 170.0]	<0.001
HDL-C, mg/dl	46.0 [39.0, 56.0]	55.0 [45.0, 66.0]	<0.001
Total cholesterol, mg/dl	187.0 [161.0, 215.0]	192.0 [166.0, 219.0]	<0.001
LDL-C, mg/dl	106.0 [82.6, 130.8]	107.2 [85.8, 131.4]	<0.001

Values are mean ± SD, median [IQR] or *n* (%).

SBP, systolic blood pressure; DBP, diastolic blood pressure; BMI, body mass index [weight in kilograms divided by the square of height in meters (kg/m^2^)]; DII, dietary inflammation index; SES, socioeconomic status; CVD, cardiovascular disease; HDL-C, high-density lipoprotein cholesterol; LDL-C, low-density lipoprotein cholesterol; mg/dl, milligrams per deciliter; g/L, grams per liter; mmHg, millimeters of mercury.

### Gender differences in mortality rates and hazard ratios: general population and population with CVD analysis

**Figure 3:**
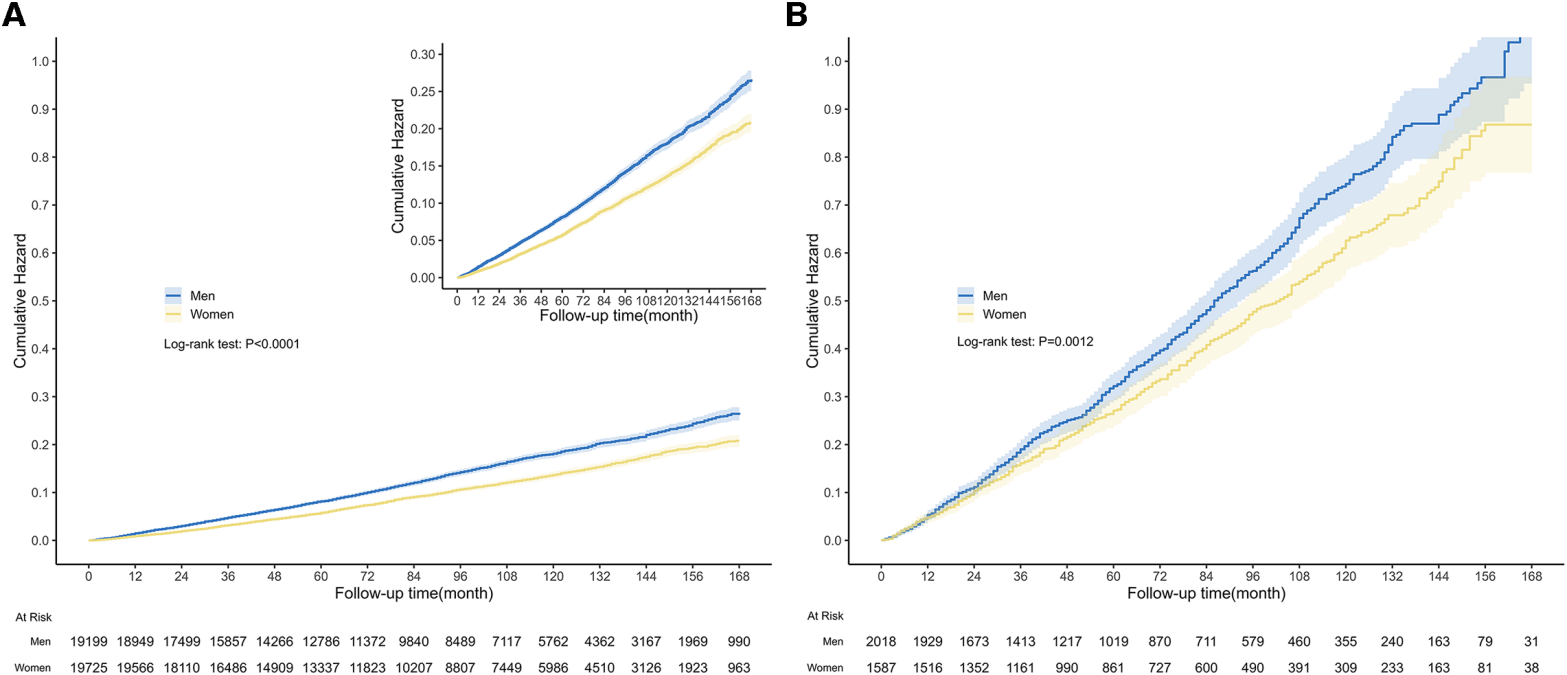
Kaplan-Meier survival curves among general population and population with CVD. The graph displayed the cumulative risk of all-cause death in the general population (**A**) and population with CVD (**B**), for both men and women. (**A**) The median follow-up length was 87 months (IQR: 47–128), during which there were 4,482 all-cause deaths in the general population. The curve illustrated that men had a significantly greater risk of all-cause death compared to women (Log-rank test: *P *< 0.0001). (**B**) And there were 1,381 all-cause deaths in the population with CVD. Similarly, men had a higher risk of all-cause death than women (Log-rank test: *P *= 0.0012).

**Figure 4:**
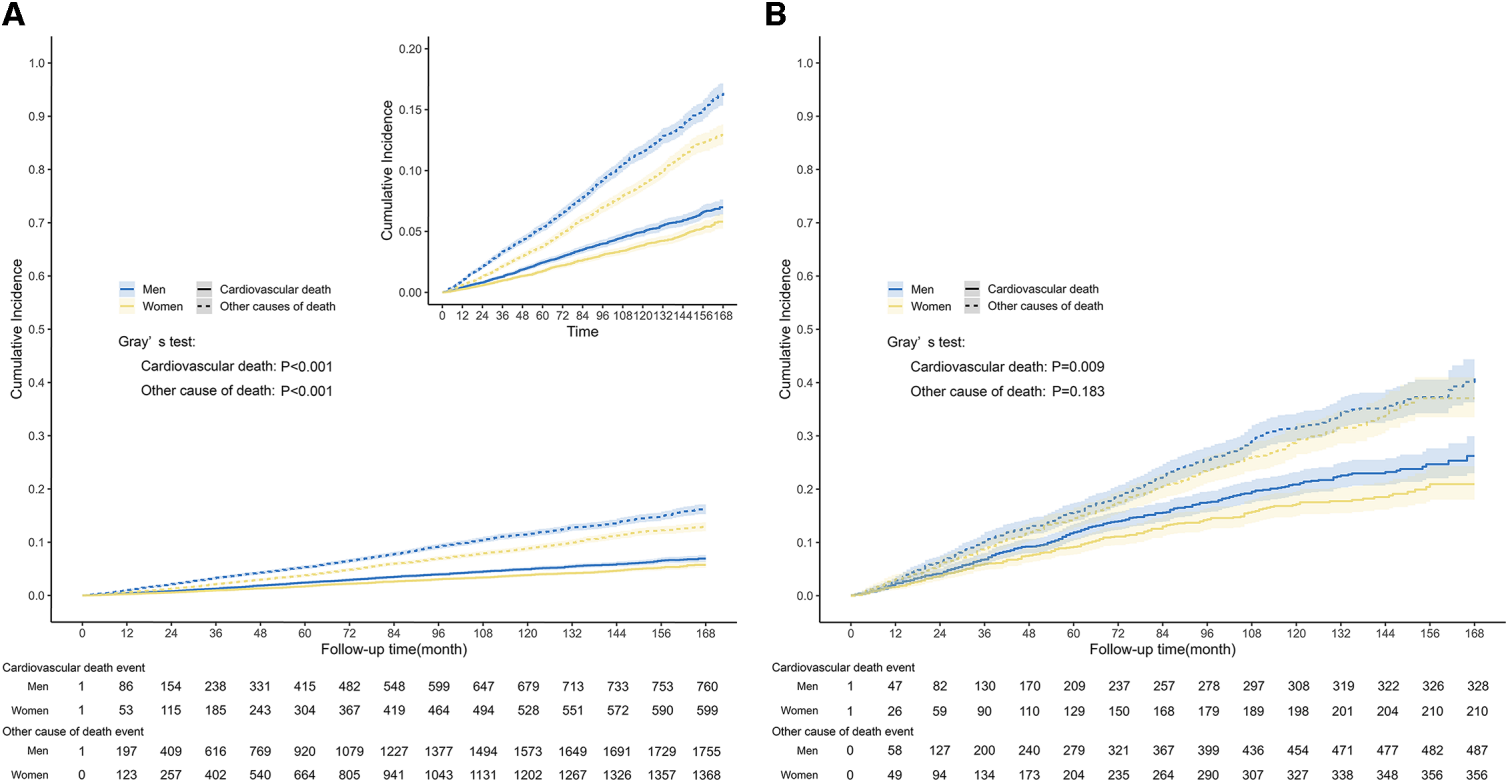
Competing-risk survival curves among general population and population with CVD. The graph depicted the cumulative incidence of cardiovascular death event and other causes of death event in the general population (**A**) and population with CVD (**B**), stratified by gender. (**A**) The median follow-up length was 87 months (IQR: 47–128), during which time a total of 1,359 cardiovascular death events occurred in the general population. The curve indicated that men had a significantly higher risk of cardiovascular and non-cardiovascular causes of death events compared to women (Gray's test: *P *< 0.001). (**B**) There were 538 cardiovascular deaths in the population with CVD. There was a significant difference in the risk of cardiovascular death events between men and women (Gray's test: *P *= 0.009), while there was no statistically significant difference in the risk of death events from other causes (Gray's test: *P *= 0.183).

During the median follow-up length of 87 months (IQR: 47–128), a total of 4,482 all-cause deaths occurred in the entire population, and among them, 1,359 deaths were related to CVD. Among people with CVD, 1,381 all-cause deaths were reported, and 538 of those deaths were attributed to CVD. The Kaplan-Meier survival curve demonstrated that men had a significantly higher risk of all-cause mortality compared to women, both in the general population (Figure 3A, *P *< 0.0001) and in the population with CVD (Figure 3B, *P *= 0.0012). In the competing risk model for cardiovascular death events, significant differences were observed between men and women in the general population. Men had a higher risk of cardiovascular death compared to women (Figure 4A, *P* for cardiovascular death < 0.001). Additionally, there were significant differences between men and women in terms of death from other causes (non-cardiovascular) (Figure 4A, *P* for other causes of death < 0.001). In the population with CVD, there was a significant difference in the risk of cardiovascular death events between men and women (Figure 4B, *P* for cardiovascular death = 0.009). However, there was no statistically significant difference in the risk of death events from other causes (non-cardiovascular) between men and women (Figure 4B, *P* for other causes of death = 0.183).

In the general population, men had higher rates of all-cause death (1.78 per 100 person-years) and cardiovascular death (0.54 per 100 person-years) compared to women (1.34 and 0.41 per 100 person-years, respectively). Tests for multicollinearity were nonsignificant. The unadjusted men: women hazard ratios (HRs) for all-cause and cardiovascular mortality were 1.33 (95% CI: 1.25 to 1.41) and 1.31 (95% CI: 1.18 to 1.46). After adjusting for confounders, the risk disparity between men and women continued to widen. Men remained significantly associated with higher risk of all-cause mortality (HR: 1.58, 95% CI: 1.48 to 1.68) and cardiovascular mortality (HR: 1.60, 95% CI: 1.43 to 1.80). The analysis confirmed that the PH assumption was satisfied.

Among individuals with CVD, the rates of all-cause mortality were 21.79 and 21.42 per 100 person-years for men and women, respectively. The rates of cardiovascular mortality were 22.59 and 22.61 per 100 person-years for men and women, respectively. The unadjusted men: women HRs for all-cause mortality were 1.19 (95% CI: 1.07 to 1.33). After adjusting for confounders, the fully adjusted HR for all-cause mortality increased to 1.34 (95% CI: 1.20 to 1.51). For cardiovascular mortality, the unadjusted and fully adjusted HRs were 1.29 (95% CI: 1.09 to 1.54) and 1.52 (95% CI: 1.26 to 1.83). For detailed information, please refer to Table 2.

**Table 2:** Hazard ratios for all-cause and cardiovascular mortality comparing men with Women: the United States, 2005–2018.

Population	Cause of death	Total number of events		Mortality rate per 100 person-years		Men *vs.* women, HR (95% CI)	
		Men	Women	Men	Women	Unadjusted^d^	Fully adjusted^e^
General population	All-cause death^b^	2,515	1,967	1.78	1.34	1.33 (1.25–1.41)	1.58 (1.48–1.68)
	Cardiovascular death^c^	760	599	0.54	0.41	1.31 (1.18–1.46)	1.60 (1.43–1.80)
Population with CVD^a^	All-cause death	815	566	21.79	21.42	1.19 (1.07–1.33)	1.34 (1.20–1.51)
	Cardiovascular death	328	210	22.59	22.61	1.29 (1.09–1.54)	1.52 (1.26–1.83)

^a^ The population with CVD refers to people with CVD, including myocardial infarction, stroke, and heart failure.

^b^ All-cause death was due to heart diseases, malignant neoplasms, chronic lower respiratory diseases, accidents, cerebrovascular diseases, Alzheimer's disease, diabetes mellitus, influenza and pneumonia, nephritis, nephrotic syndrome, nephrosis or other causes of death.

^c^ Cardiovascular death referred to death from heart diseases or cerebrovascular diseases.

^d^ Unadjusted model: only included adjustment for gender.

^e^ Fully adjusted model: included gender, confounders (age, race), and mediators (marital status, SES, lifestyle level, smoking status, CVD, hypertension, diabetes and cancer).

**Figure 5:**
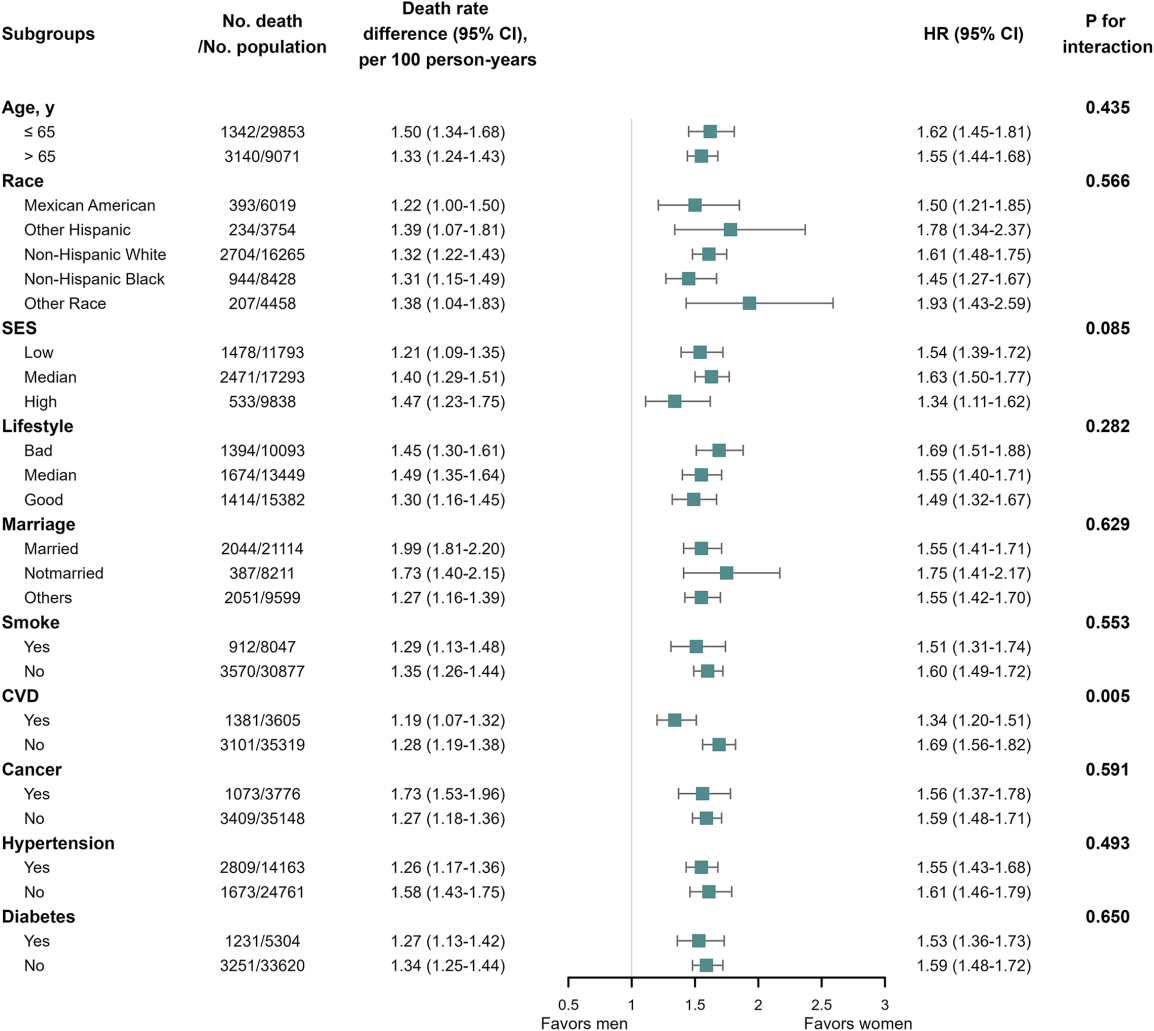
All-cause mortality among the general population in subgroup analysis. We performed subgroup analysis in the general population with all-cause mortality as the outcome, calculating the number of deaths, gender differences in mortality per 100 person-years, and the interaction of subgroup variables with gender. We only observed significant interactions between gender and CVD (*P* for interaction = 0.005) in relation to all-cause mortality outcomes.

In the subgroup analysis of the general population, a significant interaction was observed between gender and CVD (*P* for interaction = 0.005) in relation to all-cause mortality outcomes (Figure 5). Specifically, the men: women relative risk of all-cause mortality was higher in participants without prior history of CVD. In the subgroup analysis of the population with CVD, we observed an interaction between gender and SES (Supplementary Figure S3; *P* for interaction = 0.044), as well as age (Supplementary Figure S3; *P* for interaction = 0.033).

### Mediation analysis comparing men: women all-cause mortality

To investigate the underlying causes of gender differences in mortality, mediation analysis was conducted in the general population. The PM for each mediator was presented in Table 3. Uric acid was identified as a significant mediator of the association between gender and all-cause mortality, accounting for 17.4% (95% CI: 11.0% to 23.7%) of the relationship. Smoking was also identified as a key mediator, explaining 11.3% (95% CI: 9.0% to 13.6%) of the gender disparity. Furthermore, CVD was found to have a mediation rate of <10% (PM: 5.1%, 95% CI: 3.9% to 6.3%), indicating that it may be a potential mediator but not a significant one in explaining gender differences in all-cause mortality. It is important to note that certain variables showed a suppression effect, where the indirect and total effects had opposite directions. These variables included SES, lifestyle, exercise, DII, triglycerides, and albumin. As a result, the mediation proportions for these variables displayed negative values. These variables are not statistically significant, but may have practical clinical significance. More detailed information on the direct and indirect effects can be found in Supplementary Table S1. Our subgroup analysis of participants aged 65 years or older suggested that CVD (PM: 14.3%, 95% CI: 10.8% to 17.8%) and uric acid (PM: 10.4%, 95% CI: 4.8% to 16.1%) were significant mediators in explaining gender differences in mortality. These findings are in line with the primary results of this study (Supplementary Table S2).

**Table 3:** Proportion of gender differences in all-cause mortality explained by SES, lifestyle factors, biochemical indicators: the United States, 2005–2018.

Mediators	General population		Population with CVD	
	Proportion mediation, %	*P*-value	Proportion mediation, %	*P*-value
SES	−3.58 (−4.99 to −2.17)	<0.05	−5.98 (−11.19 to −0.77)	<0.05
Lifestyle	−9.13 (−12.00 to −6.26)	<0.05	−16.33 (−27.72 to −4.94)	<0.05
Smoke	11.29 (9.00 to 13.58)	<0.05	5.28 (0.82 to 9.73)	<0.05
Exercise	−15.48 (−19.28 to −11.67)	<0.05	−21.92 (−36.02 to −7.82)	<0.05
DII	−8.04 (−10.93 to −5.15)	<0.05	−10.19 (−18.41 to −1.97)	<0.05
Sleep	0.28 (−0.03 to 0.60)	>0.05	0.21 (−0.55 to 0.97)	>0.05
CVD	5.12 (3.92 to 6.32)	<0.05	NA	NA
Cancer	−0.80 (−1.33 to −0.26)	<0.05	−0.75 (−2.61 to 1.11)	>0.05
Hypertension	−0.42 (−0.79 to −0.04)	<0.05	−0.17 (−2.12 to 1.78)	>0.05
Diabetes	1.27 (0.65 to 1.89)	<0.05	0.12 (−2.76 to 2.99)	>0.05
Uric acid	17.53 (11.09 to 23.97)	<0.05	27.47 (14.26 to 40.69)	<0.05
Triglycerides	−2.84 (−5.21 to −0.48)	<0.05	−0.99 (−3.22 to 1.24)	>0.05
Albumin	−25.96 (−31.84 to −20.08)	<0.05	−18.91 (−30.89 to −6.93)	<0.05
HDL-C	1.34 (−3.18 to 5.86)	>0.05	−6.06 (−17.96 to 5.84)	>0.05
LDL-C	0.74 (0.19 to 1.29)	<0.05	0.52 (−4.27 to 5.32)	>0.05

SES, socioeconomic status; DII, Dietary Inflammation Index; CVD, cardiovascular disease; HDL-C, high-density lipoprotein cholesterol; LDL-C, low-density lipoprotein cholesterol.

The calculation of the proportion mediation (PM) involves the formula NIE/TE.

Key mediators were defined as those with a significant mediating effect and PM greater than 10%.

In addition, we observed that gender differences in the risk of all-cause mortality may vary in subgroups with history of CVD. Therefore, we conducted mediation analysis specifically in the subgroups of people with CVD, as shown in Table 3. Uric acid was found to be a key mediator in the relationship between gender and all-cause death in the CVD subgroup, with a mediation proportion of 27.5% (95% CI: 14.3% to 40.7%).

## Discussion

In this study, we discovered that the inherent disparity in mortality risk between men and women is greater than what is actually observed, both in the entire population or the population with CVD from NHANES 2005–2018. This disparity can be attributed to a combination of social and biological factors. Furthermore, the study quantified the extent to which these factors mediated the gender differences in all-cause mortality.

Previous studies have established that gender disparities in mortality are influenced by social, behavioral, and epidemiological factors (6, 35). In this particular study, we specifically examined the United States, a country characterized by notable socioeconomic disparities. Consistent with previous findings, significant gender differences in mortality rates were found within the U.S. population (36–38). Addressing and reducing the gender mortality gap is of utmost importance in promoting equitable access to evidence-based healthcare and preventive measures. This has substantial implications for clinical practice and disease prevention, particularly within the framework of precision medicine.

Due to the protective effects of estrogen, women often receive a diagnosis of CVD around 10 years later than men (4, 5). However, after menopause, the risk of developing CVD increases for women, independent of age and other cardiovascular risk factors (39). Although men have a higher absolute risk of all-cause and cardiovascular death, they tend to develop CVD at an earlier age than women and receive more timely treatment, which may benefit them more. In order to explore the gender differences in mortality, we conducted subgroup analysis in the general population. The findings revealed that once diagnosed with CVD, women had worse outcomes compared to men, which narrowed the gender gap in mortality (36). This may be attributed to the fact that women tend to be older at the onset of CVD and may delay seeking medical care, resulting in worse outcomes (40, 41). Therefore, it is crucial to provide targeted secondary prevention measures for women.

The disparities in mortality based on SES have been extensively researched and well-documented (42, 43). Our study revealed a significant gender gap in all-cause mortality among individuals with low and moderate SES in population with CVD. It is worth noting that the CVD group itself may be associated with various unhealthy lifestyle factors, including a higher prevalence of smoking and alcohol abuse behaviors (44, 45), increased obesity rates, and a greater likelihood of having concurrent comorbidities such as metabolic syndrome (46–48). These characteristics are particularly prominent in the population with CVD with low SES. The combination of unhealthy lifestyles, lack of awareness about their own health and insufficient social security exposes individuals in this group to a higher risk of death, thereby exacerbating the gender gap in mortality. In order to promote greater equality in SES and reduce the disparities in mortality between different SES classes, interventions should be specifically targeted towards individuals with CVD in the low and median SES groups (49).

To investigate the underlying causes of gender difference in mortality, we included several serological markers, including albumin, uric acid, triglycerides, LDL-C and HDL-C, in the mediation analysis. Our findings of this study support the proactive promotion of smoking cessation interventions and the importance of controlling uric acid levels to potentially reduce the gender disparity in mortality risk for both men and women. Several relevant studies have reported that elevated levels of uric acid are independent risk factor for coronary heart disease, hypertension, heart failure, and stroke (50–53). We conducted a more in-depth investigation into the interaction between gender and uric acid levels. Previous studies have highlighted that the threshold for uric acid in relation to all-cause mortality is 5.4 mg/dl (95% CI: 4.80–6.57) for men and 4.7 mg/dl (95% CI: 4.40–5.10) for women (54). We utilized this threshold to define high uric acid levels and discovered a significant interaction between gender and uric acid levels (*P *= 0.025). For individuals with uric acid levels above this defined threshold, the risk of all-cause death was 1.45-fold higher in men than in women (95% CI: 1.34–1.57). In individuals with uric acid levels below this threshold, men had a higher risk of all-cause mortality (HR = 1.84; 95% CI: 1.65–2.05). This may be due to the fact that high uric acid is more harmful in women at the same uric acid level, narrowing the gender gap in all-cause mortality. Previous studies have shown that uric acid is independently associated with fatal myocardial infarction, particularly in women (55). In addition, uric acid levels were positively correlated with cardiovascular death risk, with a stronger correlation in women compared to men (56). Our findings align with previous studies conducted in Italian populations, and it is necessary to conduct further studies in the US population to investigate suitable thresholds that can effectively differentiate between various risk groups.

Our study has several limitations. Firstly, SES and lifestyle information were primarily self-reported and recorded only once, which may have introduced measurement errors that were unavoidable. Additionally, we could not track long-term SES trajectories and post-disease lifestyle changes, as both of these factors can be influenced by disease status and thus impact study outcomes. Lastly, without medical or imaging confirmation, the accuracy of self-reported medical history may be limited. For example, a lack of information about revascularization procedures (percutaneous coronary intervention or coronary artery bypass grafting) may lead to misclassification of coronary heart disease cases.

## Conclusion

In this study, it was observed that men exhibited a higher risk of all-cause and cardiovascular mortality compared to women, within both the general population and the population afflicted by CVD. While men seemed to possess certain protective factors, such as lifestyle choices and social status, the disparities in all-cause mortality persisted even after accounting for these factors. Hence, we posit that the variations in all-cause mortality between men and women may be more pronounced at a biological or genetic level. Uric acid levels were identified as a pivotal factor mediating the gender-based disparities in all-cause mortality.

## Data Availability

The raw data supporting the conclusions of this article will be made available by the authors, without undue reservation.
