# Supplementary material for: Gender differences in all-cause and cardiovascular mortality among US adults: from NHANES 2005–2018
Source: Front Cardiovasc Med. 2024 Feb 14;11:1283132. doi: 10.3389/fcvm.2024.1283132 (PMC10899466; doi:10.3389/fcvm.2024.1283132)
Supplement: Supplementary file 1 [file Datasheet1.docx]

**SUPPLEMENTAL MATERIAL**

This appendix has been provided by the authors to give readers additional information about their work.

**Gender Differences in All-Cause and Cardiovascular Mortality Among US Adults: From NHANES 2005-2018**

**Ying Lv^1†^, Xiaodi Cao^2†^, Kai Yu^3^, Jie Pu^1^, Zhiguo Tang^1^, Na Wei^1^, Junkui Wang^1^, Fuqiang Liu^1^, Shangjian Li^1*^**

†These two authors contributed equally to this work and share first authorship

^1^Department of Cardiology, Shaanxi Provincial People’s Hospital, Xi’an, Shaanxi, China

^2^Department of Cardiology, Jiangsu Provincial People’s Hospital and the First Affiliated Hospital of Nanjing Medical University, Nanjing, Jiangsu, China

^3^Department of Cardiology, Pucheng County Hospital, Weinan, Shaanxi, China

**List of Contents**

**Page 1: Cover page**

**Page 2: List of contents**

**Page 3-4: eTable 1**

**Page 5-6: eTable 2**

**Page 7: eFigure 1**

**Page 8: eFigure 2**

**Page 9: eFigure 3**

**eTable 1. Direct and Indirect Effect Sizes for Gender and All-cause Mortality in General Participants and those with CVD (Mediated by SES, Lifestyle Factors, Biochemical Indicators)**

| **Mediators** | **General Population** | | **CVD Population** | |
| --- | --- | --- | --- | --- |
|  | HR (95% CI) | *P*-value | HR (95% CI) | *P*-value |
| **SES** |  |  |  |  |
| NDE | 0.42 (0.36 to 0.49) | <0.05 | 0.26 (0.15 to 0.38) | <0.05 |
| NIE | -0.01 (-0.02 to -0.01) | <0.05 | -0.01 (-0.02 to -0.00) | <0.05 |
| TE | 0.41 (0.35 to 0.47) | <0.05 | 0.25 (0.14 to 0.36) | <0.05 |
| PM, % | -3.58 (-4.99 to -2.17) | <0.05 | -5.98 (-11.19 to -0.77) | <0.05 |
| **Lifestyle** |  |  |  |  |
| NDE | 0.42 (0.36 to 0.49) | <0.05 | 0.26 (0.15 to 0.38) | <0.05 |
| NIE | -0.03 (-0.04 to -0.02) | <0.05 | -0.03 (-0.05 to -0.02) | <0.05 |
| TE | 0.39 (0.33 to 0.46) | <0.05 | 0.23 (0.12 to 0.34) | <0.05 |
| PM, % | -9.13 (-12.00 to -6.26) | <0.05 | -16.33 (-27.72 to -4.94) | <0.05 |
| **Smoke** |  |  |  |  |
| NDE | 0.42 (0.36 to 0.49) | <0.05 | 0.26 (0.15 to 0.38) | <0.05 |
| NIE | 0.04 (0.04 to 0.05) | <0.05 | 0.01 (0.00 to 0.02) | >0.05 |
| TE | 0.47 (0.41 to 0.53) | <0.05 | 0.28 (0.16 to 0.39) | <0.05 |
| PM, % | 11.29 (9.00 to 13.58) | <0.05 | 5.28 (0.82 to 9.73) | >0.05 |
| **Exercise** |  |  |  |  |
| NDE | 0.46 (0.40 to 0.52) | <0.05 | 0.31 (0.19 to 0.42) | <0.05 |
| NIE | -0.05 (-0.06 to -0.04) | <0.05 | -0.05 (-0.07 to -0.03) | <0.05 |
| TE | 0.41 (0.35 to 0.47) | <0.05 | 0.26 (0.14 to 0.37) | <0.05 |
| PM, % | -15.48 (-19.28 to -11.67) | <0.05 | -21.92 (-36.02 to -7.82) | <0.05 |
| **DII** |  |  |  |  |
| NDE | 0.46 (0.40 to 0.52) | <0.05 | 0.31 (0.19 to 0.42) | <0.05 |
| NIE | -0.03 (-0.04 to -0.02) | <0.05 | -0.02 (-0.04 to -0.01) | <0.05 |
| TE | 0.43 (0.37 to 0.49) | <0.05 | 0.28 (0.17 to 0.39) | <0.05 |
| PM, % | -8.04 (-10.93 to -5.15) | <0.05 | -10.19 (-18.41 to -1.97) | <0.05 |
| **Sleep** |  |  |  |  |
| NDE | 0.46 (0.40 to 0.52) | <0.05 | 0.31 (0.19 to 0.42) | <0.05 |
| NIE | 0.00 (-0.00 to 0.00) | >0.05 | 0.00 (-0.00 to 0.00) | >0.05 |
| TE | 0.46 (0.40 to 0.52) | <0.05 | 0.31 (0.19 to 0.42) | <0.05 |
| PM, % | 0.28 (-0.03 to 0.60) | >0.05 | 0.21 (-0.55 to 0.97) | >0.05 |
| **CVD** |  |  |  |  |
| NDE | 0.42 (0.36 to 0.49) | <0.05 | NA | NA |
| NIE | 0.02 (0.01 to 0.02) | <0.05 | NA | NA |
| TE | 0.44 (0.38 to 0.50) | <0.05 | NA | NA |
| PM, % | 5.12 (3.92 to 6.32) | <0.05 | NA | NA |
| **Cancer** |  |  |  |  |
| NDE | 0.42 (0.36 to 0.49) | <0.05 | 0.26 (0.15 to 0.38) | <0.05 |
| NIE | -0.00 (-0.00 to -0.00) | <0.05 | -0.00 (-0.01 to 0.00) | >0.05 |
| TE | 0.42 (0.36 to 0.48) | <0.05 | 0.26 (0.15 to 0.37) | <0.05 |
| PM, % | -0.80 (-1.33 to -0.26) | <0.05 | -0.75 (-2.61 to 1.11) | >0.05 |
| **Hypertension** |  |  |  |  |
| NDE | 0.42 (0.36 to 0.49) | <0.05 | 0.26 (0.15 to 0.38) | <0.05 |
| NIE | -0.00 (-0.00 to -0.00) | <0.05 | -0.00 (-0.00 to 0.00) | >0.05 |
| TE | 0.42 (0.36 to 0.48) | <0.05 | 0.26 (0.15 to 0.38) | <0.05 |
| PM, % | -0.42 (-0.79 to -0.04) | <0.05 | -0.17 (-2.12 to 1.78) | >0.05 |
| **Diabetes** |  |  |  |  |
| NDE | 0.42 (0.36 to 0.49) | <0.05 | 0.26 (0.15 to 0.38) | <0.05 |
| NIE | 0.00 (0.00 to 0.01) | <0.05 | 0.00 (-0.01 to 0.01) | >0.05 |
| TE | 0.43 (0.37 to 0.49) | <0.05 | 0.26 (0.15 to 0.38) | <0.05 |
| PM, % | 1.27 (0.65 to 1.89) | <0.05 | 0.12 (-2.76 to 2.99) | >0.05 |
| **Uric acid** |  |  |  |  |
| NDE | 0.38 (0.32 to 0.44) | <0.05 | 0.21 (0.10 to 0.33) | <0.05 |
| NIE | 0.06 (0.04 to 0.09) | <0.05 | 0.07 (0.04 to 0.10) | <0.05 |
| TE | 0.44 (0.38 to 0.51) | <0.05 | 0.28 (0.17 to 0.39) | <0.05 |
| PM, % | 17.53 (11.09 to 23.97) | <0.05 | 27.47 (14.26 to 40.69) | <0.05 |
| **Triglycerides** |  |  |  |  |
| NDE | 0.42 (0.36 to 0.49) | <0.05 | 0.26 (0.15 to 0.38) | <0.05 |
| NIE | -0.01 (-0.02 to -0.00) | <0.05 | -0.00 (-0.01 to 0.00) | >0.05 |
| TE | 0.42 (0.35 to 0.48) | <0.05 | 0.26 (0.15 to 0.37) | <0.05 |
| PM, % | -2.84 (-5.21 to -0.48) | <0.05 | -0.99 (-3.22 to 1.24) | >0.05 |
| **Albumin** |  |  |  |  |
| NDE | 0.45 (0.39 to 0.51) | <0.05 | 0.29 (0.18 to 0.41) | <0.05 |
| NIE | -0.08 (-0.10 to -0.07) | <0.05 | -0.04 (-0.06 to -0.02) | <0.05 |
| TE | 0.37 (0.31 to 0.44) | <0.05 | 0.25 (0.14 to 0.37) | <0.05 |
| PM, % | -25.96 (-31.84 to -20.08) | <0.05 | -18.91 (-30.89 to -6.93) | <0.05 |
| **HDL-C** |  |  |  |  |
| NDE | 0.42 (0.36 to 0.48) | <0.05 | 0.28 (0.16 to 0.39) | <0.05 |
| NIE | 0.00 (-0.01 to 0.02) | >0.05 | -0.01 (-0.04 to 0.01) | >0.05 |
| TE | 0.42 (0.36 to 0.49) | <0.05 | 0.26 (0.15 to 0.38) | <0.05 |
| PM, % | 1.34 (-3.18 to 5.86) | >0.05 | -6.06 (-17.96 to 5.84) | >0.05 |
| **LDL-C** |  |  |  |  |
| NDE | 0.41 (0.35 to 0.47) | <0.05 | 0.26 (0.15 to 0.38) | <0.05 |
| NIE | 0.00 (0.00 to 0.00) | <0.05 | 0.00 (-0.01 to 0.01) | >0.05 |
| TE | 0.41 (0.35 to 0.48) | <0.05 | 0.26 (0.15 to 0.38) | <0.05 |
| PM, % | 0.74 (0.19 to 1.29) | <0.05 | 0.52 (-4.27 to 5.32) | >0.05 |

NDE: natural direct effect; NIE: natural indirect effect; PM: proportion mediation; TE: total effect

**eTable 2. Direct and Indirect Effect Sizes for Gender and All-cause Mortality in General Participants older than 65 years (Mediated by SES, Lifestyle Factors, Biochemical Indicators)**

| **Mediators** | **HR (95% CI)** | ***P*-value** |
| --- | --- | --- |
| **SES** |  |  |
| NDE | 0.40 (0.33 to 0.47) | <0.05 |
| NIE | -0.01 (-0.02 to -0.01) | <0.05 |
| TE | 0.39 (0.32 to 0.46) | <0.05 |
| PM, % | -3.52 (-5.45 to -1.59) | <0.05 |
| **Lifestyle** |  |  |
| NDE | 0.40 (0.33 to 0.47) | <0.05 |
| NIE | -0.02(-0.03 to -0.01) | <0.05 |
| TE | 0.38 (0.30 to 0.45) | <0.05 |
| PM, % | -7.50 (-10.54 to -4.46) | <0.05 |
| **Smoke** |  |  |
| NDE | 0.40 (0.33 to 0.47) | <0.05 |
| NIE | 0.03 (0.02 to 0.03) | <0.05 |
| TE | 0.43 (0.35 to 0.50) | <0.05 |
| PM, % | 7.33 (4.69 to 9.97) | <0.05 |
| **Exercise** |  |  |
| NDE | 0.43 (0.36 to 0.51) | <0.05 |
| NIE | -0.04 (-0.05 to -0.03) | <0.05 |
| TE | 0.39 (0.32 to 0.47) | <0.05 |
| PM, % | -12.94 (-17.42 to -8.47) | <0.05 |
| **DII** |  |  |
| NDE | 0.43 (0.36 to 0.51) | <0.05 |
| NIE | -0.02 (-0.03 to -0.01) | <0.05 |
| TE | 0.41 (0.34 to 0.49) | <0.05 |
| PM, % | -6.74 (-9.58 to -3.91) | <0.05 |
| **Sleep** |  |  |
| NDE | 0.43 (0.36 to 0.51) | <0.05 |
| NIE | 0.00 (0.00 to 0.00) | >0.05 |
| TE | 0.43 (0.36 to 0.51) | <0.05 |
| PM, % | 0.03 (-0.19 to 0.25) | >0.05 |
| **CVD** |  |  |
| NDE | 0.40 (0.33 to 0.47) | <0.05 |
| NIE | 0.05 (0.04 to 0.07) | <0.05 |
| TE | 0.45 (0.38 to 0.53) | <0.05 |
| PM, % | 14.28 (10.79 to 17.76) | <0.05 |
| **Cancer** |  |  |
| NDE | 0.40 (0.33 to 0.47) | <0.05 |
| NIE | 0.01 (0.00 to 0.01) | <0.05 |
| TE | 0.41 (0.33 to 0.48) | <0.05 |
| PM, % | 1.63 (0.42 to 2.83) | <0.05 |
| **Hypertension** |  |  |
| NDE | 0.40 (0.33 to 0.47) | <0.05 |
| NIE | 0.00 (-0.01 to 0.01) | >0.05 |
| TE | 0.40 (0.33 to 0.47) | <0.05 |
| PM, % | 0.09 (-1.70 to 1.88) | >0.05 |
| **Diabetes** |  |  |
| NDE | 0.40 (0.33 to 0.47) | <0.05 |
| NIE | 0.01 (0.01 to 0.02) | <0.05 |
| TE | 0.41 (0.34 to 0.49) | <0.05 |
| PM, % | 3.24 (1.50 to 4.98) | <0.05 |
| **Uric acid** |  |  |
| NDE | 0.37 (0.29 to 0.44) | <0.05 |
| NIE | 0.04 (0.02 to 0.05) | <0.05 |
| TE | 0.40 (0.33 to 0.48) | <0.05 |
| PM, % | 10.41 (4.75 to 16.08) | <0.05 |
| **Triglycerides** |  |  |
| NDE | 0.40 (0.32 to 0.47) | <0.05 |
| NIE | 0.00 (0.00 to 0.00) | >0.05 |
| TE | 0.40 (0.32 to 0.47) | <0.05 |
| PM, % | 0.38 (-0.38 to 1.14) | >0.05 |
| **Albumin** |  |  |
| NDE | 0.41 (0.34 to 0.49) | <0.05 |
| NIE | -0.02 (-0.03 to -0.01) | <0.05 |
| TE | 0.40 (0.32 to 0.47) | <0.05 |
| PM, % | -5.78 (-8.51 to -3.05) | <0.05 |
| **HDL-C** |  |  |
| NDE | 0.39 (0.32 to 0.47) | <0.05 |
| NIE | 0.01 (-0.01 to 0.03) | >0.05 |
| TE | 0.40 (0.33 to 0.47) | <0.05 |
| PM, % | 1.86 (-4.06 to 7.79) | >0.05 |
| **LDL-C** |  |  |
| NDE | 0.39 (0.32 to 0.47) | <0.05 |
| NIE | 0.01 (0.00 to 0.02) | >0.05 |
| TE | 0.40 (0.33 to 0.47) | <0.05 |
| PM, % | 2.61 (-0.36 to 5.58) | >0.05 |

NDE: natural direct effect; NIE: natural indirect effect; PM: proportion mediation; TE: total effect

**eFigure 1. Latent Class Analysis of SES**


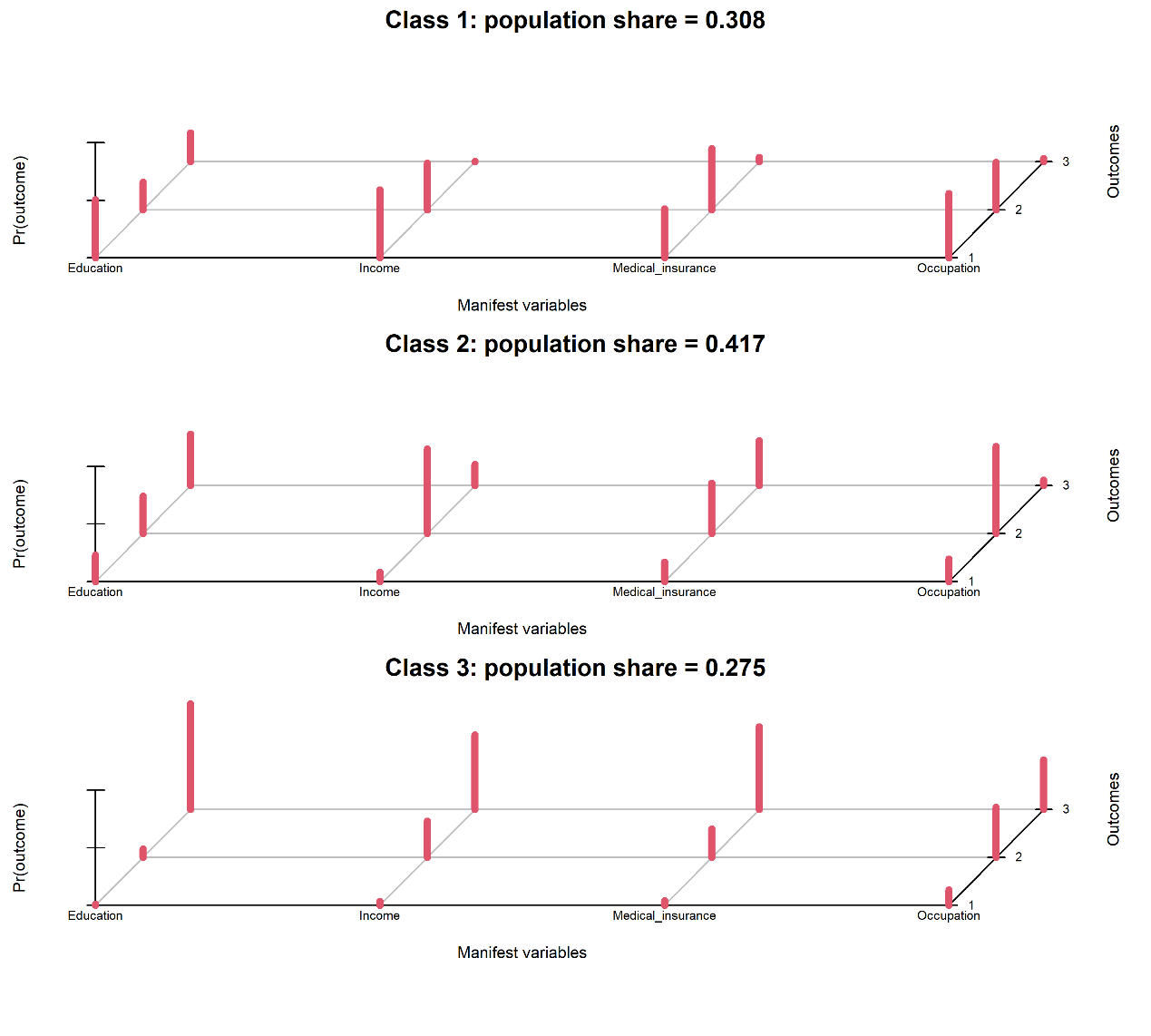


We clustered four variables: education level, income, medical insurance, and occupation. Each variable had three levels: 1 for low, 2 for medium, and 3 for high. The result of latent category analysis showed that level 1 accounted for the highest proportion of the four variables in Class 1, so Class 1 was defined as the low SES group. In Class 2, level 2 accounted for the highest proportion, so it was defined as the medium SES group. Likewise, level 3 had the highest proportion in Class 3, defined as high SES.

**eFigure 2. Latent Class Analysis of Lifestyle**


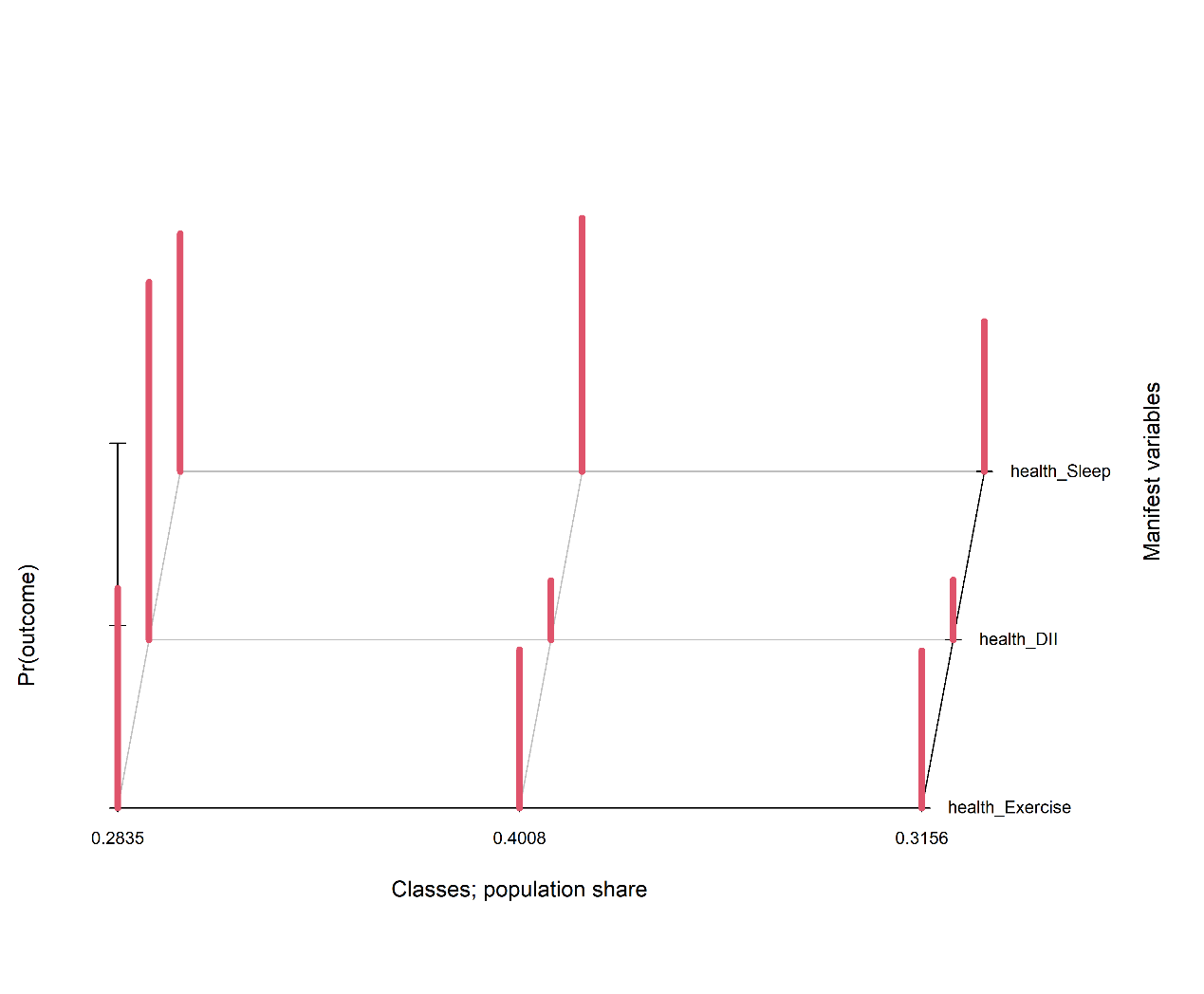


We clustered three variables: sleep, DII and exercise. Each variable had two levels: 1 for healthy, 0 for unhealthy. Level 1 accounted for the highest proportion in class 1 and was defined as healthy lifestyle. Class 2 and 3 were defined as less healthy and unhealthy lifestyle.

**eFigure 3. All-cause Mortality Among the Population with CVD in Subgroup Analysis**


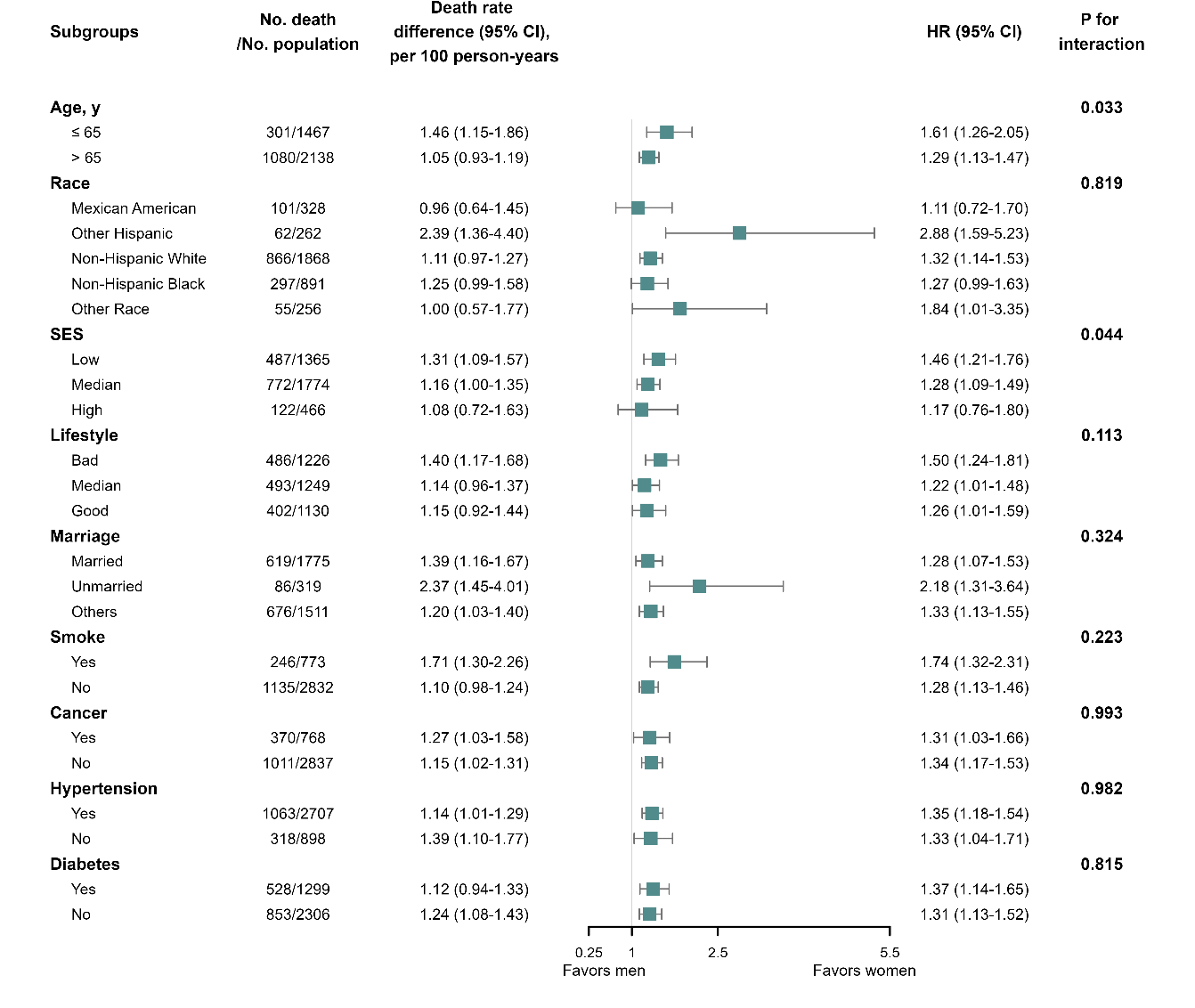


We performed subgroup analysis in the population with CVD with all-cause mortality as the outcome, calculating the number of deaths, gender differences in mortality per 100 person-years, and the interaction of subgroup variables with gender. We observed significant interactions between gender and SES (*P* for interaction=0.044) as well as age (*P* for interaction=0.033) in relation to all-cause mortality outcomes.
